# The Impact of Locoregional Treatment on Response to Nivolumab in Advanced Platinum Refractory Head and Neck Cancer: The Need Trial

**DOI:** 10.3390/vaccines8020191

**Published:** 2020-04-20

**Authors:** Andrea Botticelli, Silvia Mezi, Giulia Pomati, Paolo Sciattella, Bruna Cerbelli, Michela Roberto, Giulia Mammone, Alessio Cirillo, Alessandra Cassano, Carmela Di Dio, Alessio Cortellini, Laura Pizzuti, Graziana Ronzino, Massimiliano Salati, Patrizia Vici, Antonella Polimeni, Marco Carlo Merlano, Marianna Nuti, Paolo Marchetti

**Affiliations:** 1 Department of Clinical and Molecular Medicine, University of Rome “Sapienza”, 00161 Rome, Italy; 2 Department of Radiological, Oncological and Anatomo-Pathological Science, University of Rome “Sapienza”, 00161 Rome, Italy; 3 Department of Statistical Sciences, Sapienza University of Rome, 00161 Rome, Italy; 4 Medical Oncology, Università Cattolica del Sacro Cuore, 00168 Rome, Italy; 5 Fondazione Policlinico universitario Agostino Gemelli IRCCS, 00168 Rome, Italy; 6 Department of Biotechnology and Applied Clinical Sciences, University of L’Aquila, 67100 L’Aquila, Italy; 7 Division of Medical Oncology 2, IRCCS Regina Elena National Cancer Institute, 00144 Rome, Italy; 8 Ospedale “Vito Fazzi”, 73100 Lecce, Italy; 9 Department of Oncology and Hematology, Division of Oncology, University of Modena and Reggio Emilia, 41100 Modena, Italy; 10 Department of Oral and Maxillo Facial Sciences, Policlinico Umberto I “Sapienza” University of Rome, 00161 Rome, Italy; 11 Medical Oncology and Translational Research, Santa Croce e Carle Teaching Hospital, 12100 Cuneo, Italy; 12 Department of Experimental Medicine, University of Rome “Sapienza”, 00161 Rome, Italy

**Keywords:** squamous cell carcinoma, head and neck cancer, immunotherapy, nivolumab, locoregional treatment

## Abstract

Background: Previous locoregional treatment could affect the response to nivolumab in platinum-refractory recurrent/metastatic head and neck squamous cell carcinoma (R/M HNSCC). The aim of this study is to evaluate the impact of the clinicopathological characteristics and previous treatment in predicting early progression to nivolumab in a real-world population. Methods: This is an observational, multicenter retrospective/prospective study including patients (pts) with platinum refractory R/M HNSCC who received nivolumab 240 mg every 2 weeks from October 2018 to October 2019. We analyzed the association between previous treatment, clinicopathological characteristics, and early progression (within 3 months). Results: Data from 61 pts were reviewed. Median age was 67 years (30–82). Forty-two pts (69%) received previous locoregional treatment. Early progression to nivolumab occurred in 36 pts (59%), while clinical benefit (stable disease and partial response) was achieved in 25 pts (41%). Early progression to nivolumab was significantly associated to previous locoregional treatment both at univariate and multivariate analysis (*p* = 0.005 and *p* = 0.048, respectively). Conclusion: nivolumab in R/M HNSCC is burdened with a high early progression rate. Previous wide neck dissection and high dose radiotherapy may compromise the efficacy of nivolumab, distorting the anatomy of the local lymphatic system and hindering the priming of immune response.

## 1. Introduction

Head and neck cancer represents the sixth most common type of neoplasia worldwide with 650,000 new cases and around 330,000 deaths each year [1]. The vast majority (more than 90%) of head and neck cancer are squamous cell carcinoma arising from various subsites of the upper aerodigestive tract. These cancers are strongly associated to specific risk factors such as tobacco and alcohol intake and human papilloma virus (HPV) infection, the latter defining a distinct disease entity [2]. In the pre-immunotherapy era, the diagnosis of unresectable recurrent and/or metastatic head and neck squamous cell carcinoma (R/M HNSCC) was linked to an invariably poor prognosis with an expected survival of less than 1 year [3]. Indeed, treatment options in platinum refractory disease included monochemotherapy with taxanes and methotrexate, though none of these drugs showed a clear benefit in term of overall survival (OS) [4]. The advent of immune checkpoint inhibitors (ICIs), a class of drugs able to modulate immune suppressive pathways in order to prime an anticancer immunity, has remarkably changed the management of R/M HNSCC. In the CheckMate 141 trial [5], nivolumab, an anti-programmed death-1 (PD-1) antibody, showed a 30% reduction in the risk of death with an objective response rate of 13.3% versus 5.8% in chemotherapy arm. Following the results from this phase III study, nivolumab was approved, becoming the standard of care in platinum refractory disease. 

Past reports on immunotherapy in non-small cell lung cancer (NSCLC) and melanoma suggested a possible association between clinicopathological characteristics, previous treatment, and outcomes [6,7,8,9,10]. 

To date, only a single retrospective study evaluated the efficacy and safety of nivolumab in R/M HNSCC, showing a significant correlation between immune-related adverse events and survival [11]. However, easily-available clinical factors, such as sex, Eastern Cooperative Oncology Group performance status (ECOG PS), and metastatic site have shown to predict outcomes among patients with solid tumors treated with checkpoint inhibitors [12,13]. 

Nevertheless, the integration of immunotherapy with other treatment, including surgery, chemotherapy, or radiotherapy should be still further investigated and previous treatment could have a relevant impact on the immune response.

It was shown in a preclinical study [14] that primary tumor resection was associated with a decrease in tumor antigen presentation and T cell activity, but, at the same time, the removal of a chronic antigen exposure allows the differentiation of CD8 T cell in antitumor memory T cell. Moreover, surgery could enhance the immune response inducing the release of the antigenic load from the primary tumor. Otherwise, the differentiation of these tumor-specific CD8+ T cells into memory cells occurs in the sentinel lymph node and its surgical removal is associated with a negative outcome. Unfortunately, in HNSCC, bilateral radical neck dissection is often performed, potentially/theoretically jeopardizing the antitumor immune response. 

In addition, previous chemotherapy may modify the sensitivity to immunotherapy. Some preclinical studies have shown that a cisplatin-based regimen enhances the T cell activation, proliferation, and their cytotoxic activity, inhibiting the immunosuppressive pathways [15].

Moreover, low dose of chemotherapy, such as gemcitabine or cyclofosfamide, seems to decrease regulatory T cell (Treg) in the tumor microenvironment. These data suggest a synergistic effect of chemotherapy and immunotherapy in the treatment of solid tumors [16]. 

Furthermore, also radiotherapy could have an immune-modulatory effect even though the right dose and schedule, able to induce immunogenic death, has not been established yet, but it could be very different from the radiation plan actually used in the treatment of HNSCC [17,18].

Therefore, we conducted a retrospective multicenter study to evaluate the efficacy and safety of nivolumab in a real-world patient population with R/M HNSCC, particularly focusing on the impact of previous locoregional treatments and clinicopathological characteristics on response to immunotherapy.

## 2. Materials and Methods 

### 2.1. Patients

This is an observational, multicenter, retrospective/prospective study, including patients with platinum refractory R/M HNSCC, judged eligible for immunotherapy with nivolumab from October 2018 to October 2019. The follow-up period was from October 2018 to January 2020. 

Patients were clinically staged with contrast enhanced computed tomography (CT) scan and magnetic resonance imaging (MRI) defining baseline disease setting. Tumor tissue biopsy was performed and the diagnosis was histologically confirmed. 

Data including age, sex, body mass index (BMI), ECOG PS, comorbidities, history of tobacco smoking and alcohol abuse, primary tumor sites, previous locoregional treatment, previous chemotherapy and total platinum dose received, and histology were retrospectively collected. Inclusion criteria were: platinum refractory R/M HNSCC, treated with platinum-based chemotherapy (last platinum administration less than 6 months before), adequate liver, renal, and hematologic basal function, PS ≤ 2, no other concomitant or previous malignant disease.

Patients who had PS > 2, a primary non-squamous histology or patients with autoimmune disease were not included in the analysis. All patients provided a written informed consent, and the protocol approval of Local Ethics Committee was obtained [CE 5618]. 

### 2.2. Treatment and Assessments

Nivolumab was administered according to the standard schedule: 240 mg intravenously at 2-weeks interval until disease progression or inacceptable toxicities. 

Treatment toxicity was assessed every 2 weeks, according to the National Cancer Institute-Common Terminology Criteria for Adverse Events version 4.0 (CTCAE version 4.03, 2010). Imaging assessment with contrast enhanced CT and/or MRI was performed after 6 cycles or before in case of evident clinical disease progression. Clinical benefit (CB), which reflected the proportion of patients with complete response (CR), stable disease (SD), or partial response (PR), was assessed according to immune Response Evaluation Criteria in Solid Tumours (i-RECIST) [19]. Patients experiencing disease progression (PD) within three months from the beginning of nivolumab were defined as early progressors. Progression free survival (PFS) was defined as the time in months from the start of immunotherapy until the occurrence of progression or death or the date of last follow up. OS was the time in months from the start of immunotherapy to the date of death or last follow up. 

### 2.3. Statistical Analysis 

In the descriptive analysis, quantitative variables were described as median and range, while qualitative variables were reported as number and percentage. The association between each clinicopathological characteristic and early PD was evaluated using the univariate and multivariable logistic regression model. Statistical significance was set at *p* < 0.05. All analyses were performed using SAS 9.4 (SAS Institute Inc., Cary, NC, USA).

## 3. Results

A total of 61 patients with platinum-refractory R/M HNSCC, who received nivolumab at six Italian centers, were included in this study. Their clinic-pathological characteristics are reported in Table 1. Median age was 67 years (range, 30–82 years), and 50 patients were male (82%). Baseline ECOG PS, evaluated before the start of nivolumab, was 0, 1, and 2 in 11 (18%), 34 (55.7%), and 16 (26.2%) patients, respectively. The primary tumor site was the oropharynx in 14 patients (23%), the hypopharynx in 8 patients (13.1%), the larynx in 19 patients (31.1%), the oral cavity in 14 patients (23%), and other subsite in 6 patients (9.8%). Histological type was squamous cell carcinoma in all the patients with a tumor grade G2 and G3 in 9 and 33 patients, respectively. Grading was missing in 19 biopsies (31.2%). HPV status was negative in 11 patients and positive in 2 patients with oropharyngeal cancer. At the baseline imaging evaluation with contrast enhanced CT, 11 patients (18%) had exclusively recurrent disease without distant metastasis and 50 patients (82%) had 1 or more metastatic site. 

Previous treatment is shown in Table 1. Overall, 42 patients (69%) received a locoregional treatment including surgery and/or radiotherapy; in particular, 9 patients (14.7%) received surgery, 9 patients (14.7%) received chemoradiotherapy, 24 patients (39.3%) both surgery and chemoradiotherapy. Standard first line platinum-based chemotherapy, according to the phase III EXTREME trial [20], was administered in 53 patients (87%). Overall, 27 patients (44%) received carboplatin-based chemotherapy and underwent disease progression within 6 months from the last carboplatin dose, while 28 patients (46%) received cisplatin-based chemotherapy and underwent disease progression within 6 months from the last cisplatin dose. 

**Figure 1:**
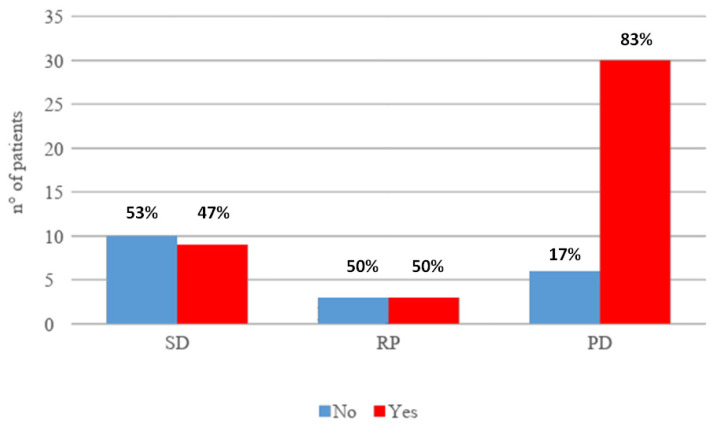
Association between response to immunotherapy and previous locoregional treatment. SD: stable disease; RP: partial response; PD: progressive disease.

Clinical benefit was achieved in 25 patients (39%); in particular 6 patients (10%) had partial response and 19 patients (31%) a stable disease. Overall, early progression occurred in 36 patients (59%). Among the patients who experienced disease progression, 30 patients (83%) vs. 6 (17%) patients received previous locoregional treatment as shown in Figure 1.

No high grade G3–G4 toxicities were reported. For all patients, median PFS and OS was 3 months (1–11 months) and 4 months (2–11 months), respectively; 30 patients (49%) were alive at the last follow-up visit. 

A possible association between early progression, clinicopathological characteristics, and previous treatment was analyzed as it is shown in Table 2. At univariate analysis, early progression to Nivolumab was significantly associated to previous locoregional treatment (*p* = 0.005) and the predictive role of previous locoregional treatment was confirmed at the multivariate analysis (*p* = 0.048). No significant association was found between early progression and each type of previous locoregional treatment (single strategy with surgery or definitive chemoradiotherapy versus both surgery and chemoradiotherapy, *p* = 0.075 versus *p* = 0.083 using the multivariate analysis, respectively). Baseline disease staging (exclusively recurrent vs. metastatic disease) was not included in the analysis (Table 2) because among the patients with only recurrent disease, only one did not experience early progression, and this would lead to estimation problems.

## 4. Discussion

In our study, previous locoregional treatments, including surgery and/or chemoradiotherapy, seem to be significantly associated with a high risk of early progression in R/M HNSCC patients treated with nivolumab. Despite nivolumab being effective and safe, the early progression rate was remarkably high. Consequently, there is an urgent need to understand how to decrease the high progression risk and improve the effectiveness of immunotherapy identifying the possible clinicopathological and/or anamnestic factors predicting response or resistance to nivolumab. Patients with locally advanced HNSCC often received complex multimodal treatment including wide neck dissection, responsible for the major distortion of the local lymphatic system, associated with high dose chemoradiotherapy, representing a possible cause of damage on the activity and vitality of local immune system cells. Although the multimodal treatment is associated with an improvement in local disease control and a relevant reduction in local recurrence, it could cause, in the long term, an irreversible damage on the ability of the local immune system to trigger a protective immune response against the disease recurrence. In vitro and in vivo studies showed that the blockade of PD-1 or PD-L1 promotes antitumor activity. The link between inhibitory immune checkpoint PD-1, often expressed on T lymphocytes, and this ligand programmed death ligand-1 (PD-L1), primes an inhibitory signal, blocking the effector and cytotoxic function of T lymphocytes. Indeed, PD1-deficient mice often developed autoimmune disease, while inhibition of PD-1/PD-L1 interaction lead to tumor regression in mice [21,22]. Moreover, the antibody mediating PD-1/PD-L1 pathway blockade promotes to an increase in effector antigen-specific T-cell count and modulates cytokines secretion in vitro and in murine models [23,24] Further recent preclinical studies have shown that surgery and radiotherapy can positively influence the effect of immunotherapy, including anti PD-1 agents, by acting as in situ vaccines, although several points have not been clarified: the right timing of immunotherapy administration, the right radiotherapy dose and fractionation, and the role of lymph nodes dissection. Conversely, the imbalance between all these elements could lead to a negative and immunosuppressive effect [14,25,26,27].

Primary tumor surgical resection can be associated with an immunosuppressive effect critically relevant in promoting escape of occult tumor cells and metastatic spread [28]. Indeed, surgery induces the release of several cytokines, chemokines, and growth factor involved in the wound healing process. At the same time, these factors promote the expansion of Tregs, myeloid-derived suppressor cells (MDSC) and macrophages M2 resulting in the impairment of NK cells activity, decreasing T cell proliferation, and increasing PD-L1 expression. All these events are peculiar to the immunosuppressive postoperative phase which can constitute an important therapeutic window in which immunotherapy could represent a promising opportunity [29,30]. At the same time, surgery can contribute to the development of anti-tumor memory cells, but this event is strictly dependent on the presence of the sentinel lymph node, that, if removed, can be associated with a negative effect on the development of the immune response [14]. In fact, while lymph nodes have a central role in immune response induction, the systemic effect of their dissection is still uncertain, resulting both in enhancement and disempowerment of the immune response in the gut system and head–neck region, respectively [31]. Additionally, radiation therapy could enhance the immunosuppressive effect deriving from surgery and, contemporarily, trigger a systemic immune response through the radiation-induced immunological cell death [32]. Indeed, radiotherapy can act as an in situ vaccine in patient with irradiated tumor, through the induction of anti-tumor T-cell response. However, the trigger of systemic immune response is strictly dependent on the dose and fraction used in the radiation therapy schedule and the cancer cell lines [33]. The high dose of radiotherapy applied in the curative management of HNSCC could not allow the development of anticancer immune response, but, instead, it could compromise, through the tissue damage, the activity of immune system cells and, like surgery, disrupt the anatomy of draining lymph nodes station. Traditional fractionated radiation is locally immunosuppressive, because lymphocytes are sensitive to radiation doses and are cleared rapidly from the radiation field [34]. The effects of surgery and radiotherapy on immune system may explain the statistically significant association between previous locoregional treatment and early progression, as it was shown at the multivariate analysis in our study.

In this study, the activity of nivolumab in R/M HNSCC was in line with results of the pivotal phase III trial [5]: in particular, response rate was 13.3% in CheckMate 141 vs. 10% of partial response in our study group. Nevertheless, our disappointing results about OS can be explained by the short follow-up period and by the high rate of early progressions. Moreover, in the CheckMate 141, G3–G4 toxicities rate was 13.1%, while no relevant toxicities were reported in our study. Consequently, nivolumab was shown to be safe also in a real-world population with unfavorable clinical characteristics. Indeed, in our group of patients, baseline ECOG PS was 1 and 2 in 55.7% and 26.2% of patients, respectively, highlighting the important frailty of patients in this setting, often heavily pre-treated and with symptomatic aggressive disease. Moreover, most of patients presented with a high disease burden at diagnosis, with 82% of them having more than one metastatic site. All these negative clinical characteristics justify, at least in part, the poor results obtained in terms of survival. A retrospective study [11], including 100 patients with R/M HNSCC treated with nivolumab, showed good results with a disease control rate of 49% and a median OS of 9.6 months, although the study population was heterogeneous in terms of primary disease subsite and histotype. The present study has some limitations to be acknowledged. This is a retrospective multicenter study with a short follow up and a relatively small sample size, thereby with potential for inherent biases. Therefore, a longer follow-up window and more patients are required to perform a reliable survival analysis.

Using a multivariate analysis, previous locoregional treatments, including surgery and/or chemoradiotherapy were confirmed to be the only independent predictive factor for early PD. This evidence suggests that immunotherapy could probably give the best advantage in other disease settings and/or combined with curative treatment modality, when neck anatomy is still inviolate. Moreover, in locally pre-treated patients (with surgery and/or radiotherapy) experiencing R/M HNSCC, immunotherapy may be less effective in disease control for induced immune system impairment. 

## 5. Conclusions

**Figure 2:**
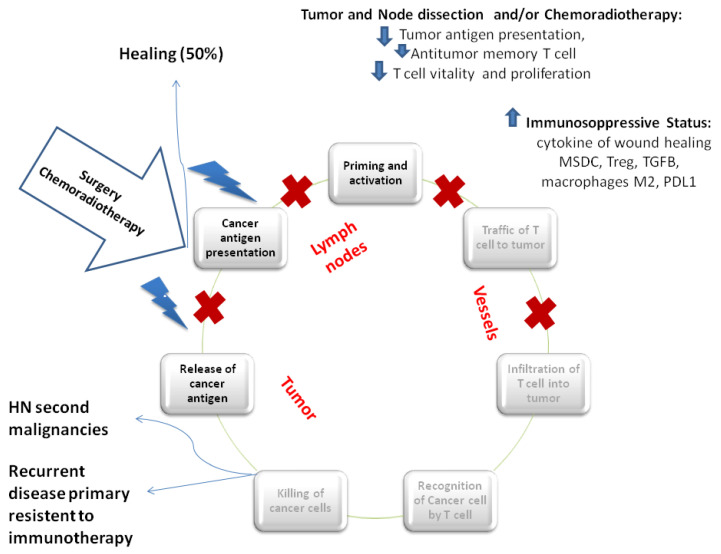
Locoregional treatment and immunity cycle. Locoregional treatment could block the immunity cycle at different level. Primary tumor resection induces a reduction in tumor antigen presentation, T cell activity, and, at the same time, the development of anti-tumor specific memory T cell which is definitively compromised by local lymph nodes dissection. The first postoperative phase is characterized by the increase of cytokines of wound healing, PD-L1 expression, myeloid-derived suppressor cells (MDSC), macrophages M2, and Treg. Similarly, radiotherapy promotes the release of cancer antigen through the induction of immunogenic cell death, increasing the antitumor T cell response and cross antigen presentation. Contemporarily, radiotherapy could promote an immunosuppressive status through the increase of local MDSC, PD-L1 expression, Treg, and macrophages M2. Healing occurs if all neoplastic cells have been radically removed after the intensive multimodal treatment and if micrometastatic disease is completely eradicated. However, head and neck second malignancies could occurr for impaired immunosurveillance. Recurrent disease with a primary induced resistance to immunotherapy is the result of an incomplete and ineffective first immune response.

Radical surgery with the demolition of the complex lymphatic network of the neck associated with adjuvant high dose radio-chemotherapy or primary radio-chemotherapy, carried out for reducing local recurrence and improve disease control, radically modify the anatomy of the local lymphatic system, hindering the priming of immune response (Figure 2). 

The effects of a previous aggressive, multimodal primary treatment on the immune system can mutilate the potential of the immune system, compromising the efficacy of nivolumab in R/M setting and may explain the association between previous locoregional treatment and high early progression rate, expression of primary resistance to ICIs treatment, as it was shown at the multivariate analysis in our study.

In conclusion, in the future, it will be necessary to further individualize therapeutic strategies based on molecular biomarkers, clinic-pathological characteristics, and previous therapeutic history, which can lead in immune system impairment, and integrate immunotherapy with standard treatment such as surgery, chemotherapy, and radiotherapy in order to improve results in term of response rate and survival.

## Figures and Tables

**Table 1:** Baseline clinicopathological characteristics.

Characteristic	All Patients N 61 (%)
Age (years)	
Median Age (range)	67 (30–82)
Gender	
Male	50 (82)
Female	11 (18)
Baseline PS ^1^ before nivolumab	
0	11 (18)
1	34(55.7)
2	16 (26.2)
Risk factors	
Smoking history	32 (52.5)
Alcohol abuse	17 (27.9)
Tumor Location	
Oral cavity	14 (23)
Oropharynx	14 (23)
Hypopharynx	8 (13.1)
Larynx	19(31.1)
Other	6 (9.8)
Histology	
Squamous Cell Carcinoma	61 (100)
Grading	
2	9 (14.8)
3	33 (54)
Missing	19 (31.2)
HPV ^2^	
Positive	2 (3.3)
Negative	11 (18)
Not reported	48 (78.7)
Recurrent Disease Metastatic site	11 (18)
	50 (82)
Previous locoregional treatment	42 (69)
Surgery	9 (14.7)
Chemoradiotherapy	9 (14.7)
Surgery and Chemoradiotherapy	24 (39.3)
First line platinum-based chemotherapy	53 (87)
Previous Carboplatin	27 (44)
Previous Cisplatin	28 (46)
Unknown	6 (10)

^1^ PS: performance status; ^2^ HPV: human papilloma virus.

**Table 2:** Correlation between clinicopathological factors and early progression during nivolumab.

Univariate Analysis			Multivariate Analysis	
Characteristic	OR ^1^ (95% CI ^2^)	*p*	OR (95% CI)	*p*
Sex				
female vs. male	3.83 (0.75–19.56)	0.106	4.82 (0.62–37.50)	0.133
Age	0.99 (0.94–1.04)	0.580	0.94 (0.87–1.02)	0.151
Alcohol history	1.39 (0.44–4.44)			
yes vs. not		0.575	1.26 (0.26–7.96)	0.775
Smoking				
Yes vs. not	0.36 (0.12–1.11)	0.075	0.32 (0.07–1.49)	0.147
Subsite				
Oropharynx	1.31 (0.32–5.43)	0.711	2.38 (0.31–18.18)	0.403
Hypopharynx	0.73 (0.14–3.82)	0.707	0.60 (0.07–4.89)	0.635
Oral cavity	1.82 (0.42–7.94)	0.427	1.42 (0.22–9.02)	0.713
Larynx	1.00 (1.00–1.00)		1.00 (1.00–1.00)	0.382
Others	0.36 (0.05–2.50)	0.303	0.27 (0.01–5.03)	
Locoregional treatment				
yes vs. not	5.42 (1.67–17.56)	0.005 ^4^	5.41 (1.02–28.74)	0.048 ^5^
Platinum-CT ^3^				
Cisplatin	0.42 (0.14–1.28)	0.127	0.31 (0.08–1.25)	0.099
Carboplatin	1.00 (1.00–1.00)		1.00 (1.00–1.00)	
Unknown	0.42 (0.07–2.55)	0.347	0.30 (0.02–4.38)	0.378

^1^ OR: odds ratio. ^2^ CI: confidential interval. ^3^ CT: chemotherapy. ^4,5^ Statistical significance was set at *p* < 0.05.
